# Colorectal cancer screening in the elderly

**DOI:** 10.1186/1471-2482-13-S1-A29

**Published:** 2013-09-16

**Authors:** R Mastromarino, A Esposito, M Candida, I Florio, C Dodaro

**Affiliations:** 1 Surgical, Anesthesiology-Rianimative and Emergency Sciences Department, Università Federico II, via Pansini 5 Napoli, Italy

## Background

Colorectal cancer is a major cause of morbidity and mortality throughout the world.

The likelihood of colorectal cancer diagnosis increases after the age of 40, increases progressively from age 40, rising sharply after age 50. The highest incidence rate is observed in persons aged ≥80 years.

Fortunately, the vast majority of cases and deaths from colorectal cancer can be prevented by applying existing knowledge about cancer prevention, so targeted screening programs, more sophisticated diagnostic procedures and early therapeutic intervention could, in time, substantially reduce the morbidity and mortality associated with colorectal cancer.

## Methods and results

The purpose of a good screening program is the discovery of the tumor at an early stage and curable for the possible removal of precancerous lesions.

Several major organizations have developed guidelines for colorectal cancer screening. Although some details of their recommendations very regarding which age to start screening program, no useful are information regarding which age to stop it.

There are some basic criteria that define the effectiveness of a screening exam.

The relevance of these criteria will change depending on the age of the patient. Even the cost-benefit ratios of screening examinations and the likelihood of benefit from therapeutic interventions early may change depending on the age of the patient.

From February 2004 to March 2012 a total of 239 patients, treated for colorectal cancer in our Department of General and Abdominal Surgery of University “Federico II” in Naples, 150 patients over 65 were diagnosed by a screening program. In terms of cost-benefits from our retrospective analysis we observed a net improvement compared with the past, and a better use of instrumental investigations for early diagnosis and stadiation of cancer.

## Conclusions

According to the clinical studies, the decision whether to interrupt or not screening of colorectal cancer should be taken on the basis of factors such as the patient's age, life expectancy, and the presence of associated diseases. Due to the effectiveness in terms cost-benefits of screening program for patients at highest risk of developing colorectal cancer should be started at a younger age.
